# Optical chemosensors for the detection of proximally phosphorylated peptides and proteins

**DOI:** 10.1039/d1cb00055a

**Published:** 2021-04-21

**Authors:** Aaron D. Cabral, Tudor B. Radu, Elvin D. de Araujo, Patrick T. Gunning

**Affiliations:** Department of Chemical and Physical Sciences, University of Toronto Mississauga 3359 Mississauga Road Mississauga Ontario L5L 1C6 Canada patrick.gunning@utoronto.ca; Department of Chemistry, University of Toronto 80 St George Street Toronto Ontario M5S 3H6 Canada

## Abstract

Proximal multi-site phosphorylation is a critical post-translational modification in protein biology. The additive effects of multiple phosphosite clusters in close spatial proximity triggers integrative and cooperative effects on protein conformation and activity. Proximal phosphorylation has been shown to modulate signal transduction pathways and gene expression, and as a result, is implicated in a broad range of disease states through altered protein function and/or localization including enzyme overactivation or protein aggregation. The role of proximal multi-phosphorylation events is becoming increasingly recognized as mechanistically important, although breakthroughs are limited due to a lack of detection technologies. To date, there is a limited selection of facile and robust sensing tools for proximal phosphorylation. Nonetheless, there have been considerable efforts in developing optical chemosensors for the detection of proximal phosphorylation motifs on peptides and proteins in recent years. This review provides a comprehensive overview of optical chemosensors for proximal phosphorylation, with the majority of work being reported in the past two decades. Optical sensors, in the form of fluorescent and luminescent chemosensors, hybrid biosensors, and inorganic nanoparticles, are described. Emphasis is placed on the rationale behind sensor scaffolds, relevant protein motifs, and applications in protein biology.

## Abstract 2

This review details optical detection methods for proximal multi-site phosphorylation, a critical post-translational modification in protein biology.

## Graphical abstract

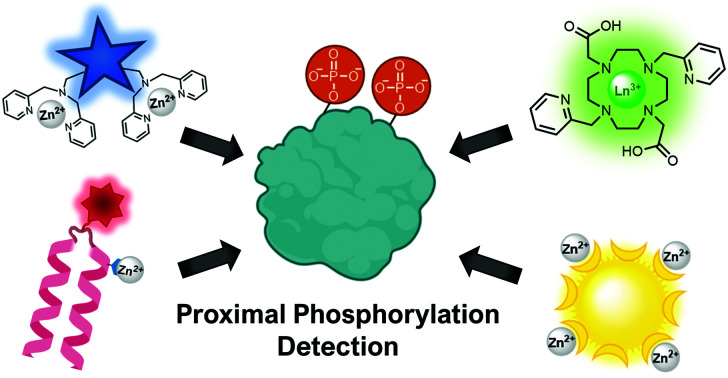

## 1. Introduction

**Fig. 1:**
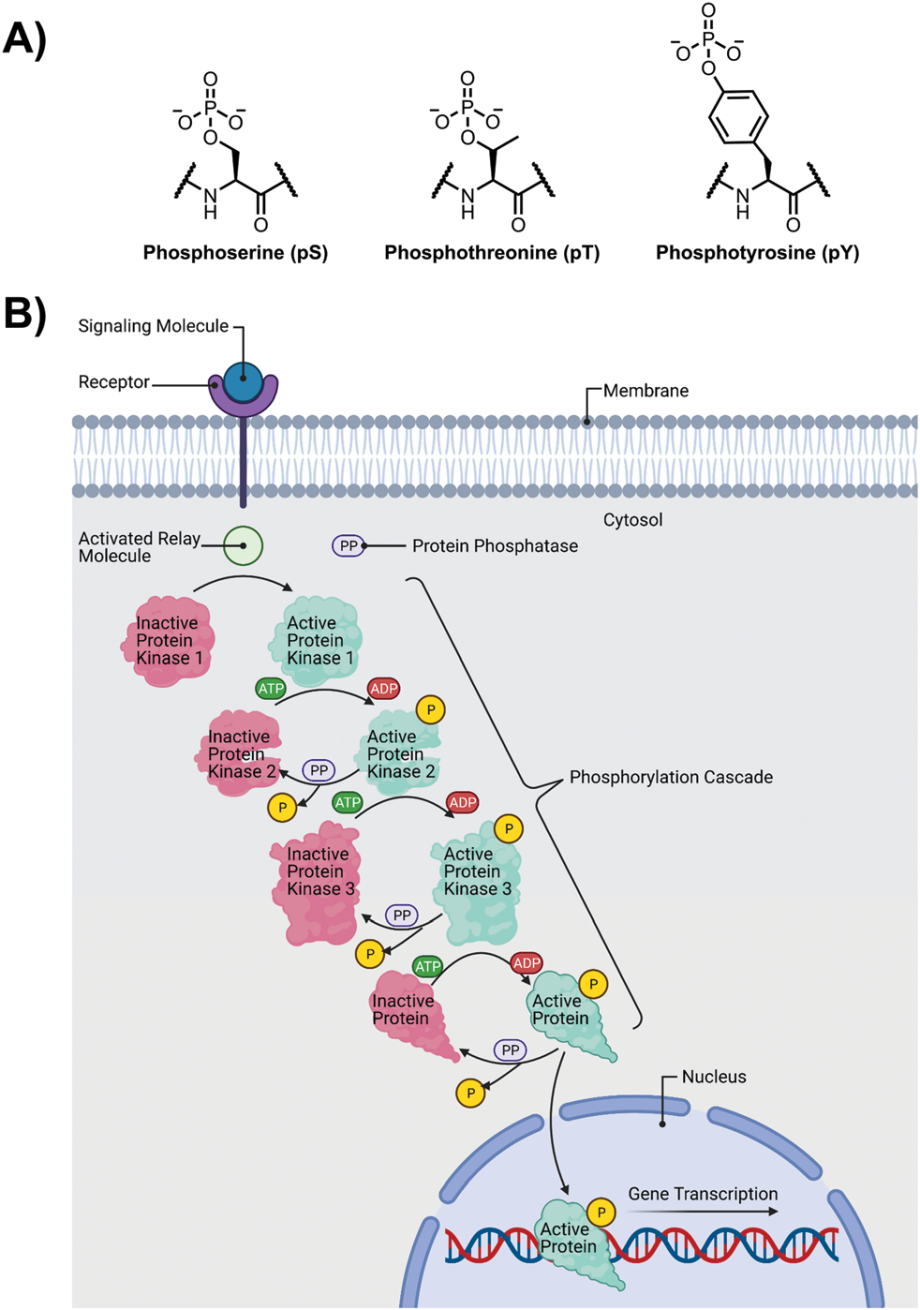
(A) Chemical structure of phosphorylated amino acids phosphoserine (pS), phosphothreonine (pT) and phosphotyrosine (pY); (B) Illustration of a representative phosphorylation/dephosphorylation signal transduction pathway.

The detection of phosphate motifs is an active field of molecular recognition research as phosphorylation is the most prevalent post-translational modification for eukaryotic proteins and has been identified on ∼65% of all proteins.^1^ Phosphorylation is a key regulator of protein activity, predominantly acting through induction of conformational changes or altering interactions with other biomolecules (Fig. 1A).^2,3^ Although phosphorylation has typically been modelled as an “on–off” switch, it has been observed that clusters of phosphosites can have cooperative, positionally-dependent and/or integrative functions.^4^ In a bioinformatics analysis of MS-confirmed phosphosites, approximately 30% of all phosphoproteins contain at least one proximal phosphosite cluster, where the majority of sites are within 4 amino acids.^4^ Indeed, phosphorylation sites have been observed to be clustered together and occupy spatially proximate regions at a higher frequency than would be expected by random chance in protein tertiary structures.^5,6^

It has been widely observed that differing phosphorylation patterns can lead to different protein interactions with distinct biological outcomes.^7^ Proximal phosphorylation has been shown to affect many cellular functions including protein localization,^8^ degradation,^9^ signaling cascades,^10,11^ gene expression,^12–14^ and the cell cycle (Fig. 1B).^15^ Multisite phosphorylation patterns have been shown to be functionally significant beyond the capability of single site modification.^16^ The anionic charged surfaces of multiple phosphosite clusters can induce large-scale conformational changes or allow for complementary binding with positively-charged binding partners.^17^ In some cases, the bulk anionic charge from a phosphosite cluster is sufficient and sequence specificity is not required.^18^ Additionally, multiple phosphorylation sites have been observed to provide overlapping or redundant levels of regulation for efficient control of protein activity.^19^ Notably, proximal phosphorylation events have a strong tendency to arise from the same kinase.^20^ These sites typically regulate similar biochemical pathways and are prone to cluster in both sequence and space.^21^ Furthermore, the levels of multiphosphorylation can also act as a feedback mechanism for downstream effects.^22^ Phosphorylation events can exhibit crosstalk with each other and with other post-translational modifications such as ubiquitination.^23,24^

Importantly, proximal phosphorylation motifs have been implicated in aberrant protein states, including the overactivation of kinases and cytotoxic protein aggregation. Proximal phosphorylation clusters have been observed to be functionally important in cancer targets such as PTEN,^25^ SIRT1,^26^ BRD4,^27^ and ERK,^28^ and other diseases such as rheumatoid arthritis (SYK).^29^ The clinically important pTau protein is hyper-phosphorylated in proximal clusters that differ in associated neurodegenerative diseases.^30^ Proximal phosphorylation is also a common modification found in intrinsically disordered regions of proteins.^31,32^ These motifs have been observed beyond eukaryotes such as in viral proteins of hepatitis C.^33^ It is postulated that phosphoprotein receptors can interrupt phosphoprotein–protein interactions as a viable medicinal chemistry strategy.^34,35^

Tandem mass spectrometry (MS/MS) is the phosphoproteomics gold standard for the identification and quantification of protein phosphorylation sites.^36^ However, MS/MS phosphoproteomic approaches have several challenges including the use of costly instrumentation coupled to highly technical sample and data processing pipelines.^37^ Additionally, the MS/MS approach requires the need for relatively large amounts of protein sample^38^ that may not be able to overcome low efficiencies for multi-phosphorylated peptides due to ion suppression effects and fragmentation,^39^ or the inherent low abundance of phosphopeptides.^40^ Several techniques enabling enrichment of proximally phosphorylated peptides have recently emerged, which dramatically aids MS/MS analysis in the separation of complex mixtures and/or increasing signal-to-noise of low abundant multi-phosphorylated peptides.^41–44^ In recent years, the introduction of highly sensitive MS hardware that is suitable for single-cell phosphoproteomics can overcome several of the challenges related to sample quality. However, researchers still incorporate complex protocols with enrichment steps to access these deep levels of the proteome.^45^ Phosphorylation state-specific antibodies are also widely used in a variety of assay types including enzyme-linked immunosorbent assays (ELISA), western blotting and immunoimaging.^46^ Many phosphorylation state-specific antibodies are commercially available, and advancements in the production process have shortened the generation time for novel antibodies to as little as 2 weeks.^47^ However, applying this strategy for proximal phosphorylation detection requires a more complex sandwich array format where multiple antibodies detect more than one phospho-site epitope on the same protein, which must be stringently validated to have little interference or non-specific binding.^46^ Other reported analytical methods for proximal phosphorylation detection include HPLC separation,^48^ impedance spectroscopy,^49^ and nanopore sequencing.^50,51^ Chemically, multi-phosphorylated peptides are also challenging to synthesize using traditional methods, which further hampers their capacity as experimental tools.^52^ However, facile and robust detection of protein phosphorylation remains a challenge for many applications due to method limitations.^53^ As such, convenient and high-throughput analytical solutions for studying protein proximal phosphorylation are in great demand.

**Fig. 2:**
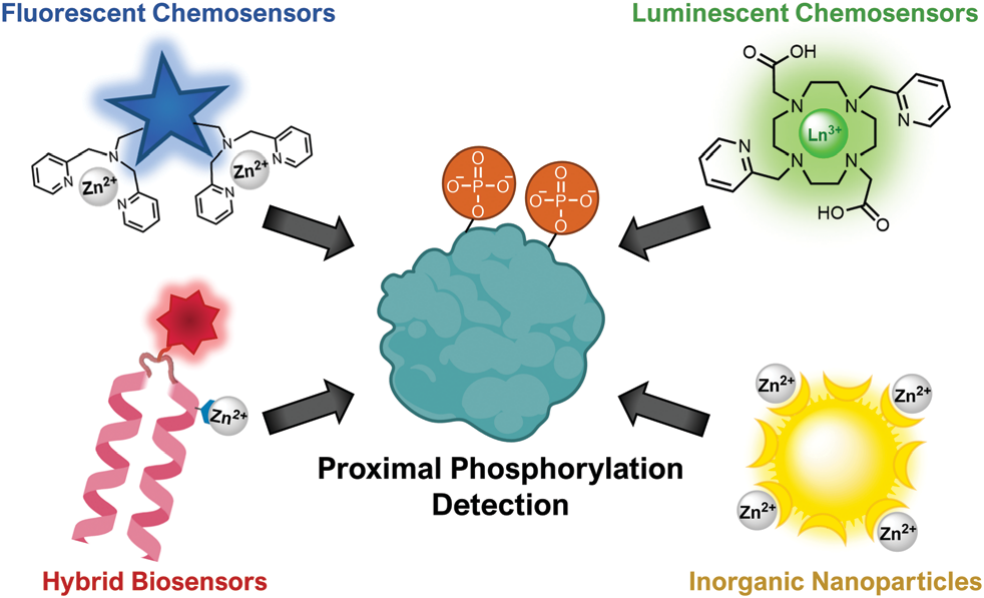
Illustration of the types of optical sensors for proximal phosphorylation detection.

This review will cover optical sensing of proximal phosphorylation on peptides and proteins. A variety of sensing strategies have been employed (Fig. 2). The focus has been on employing small-molecule fluorescent chemosensors, although luminescent sensors, hybrid biosensors and inorganic nanoparticles have also demonstrated significant success. This review will highlight the rationale behind sensor design, analyte detection limits, sequence selectivity for specific proximal motifs and their relevant biological applications.

## 2. Fluorescent chemosensors

**Fig. 3:**
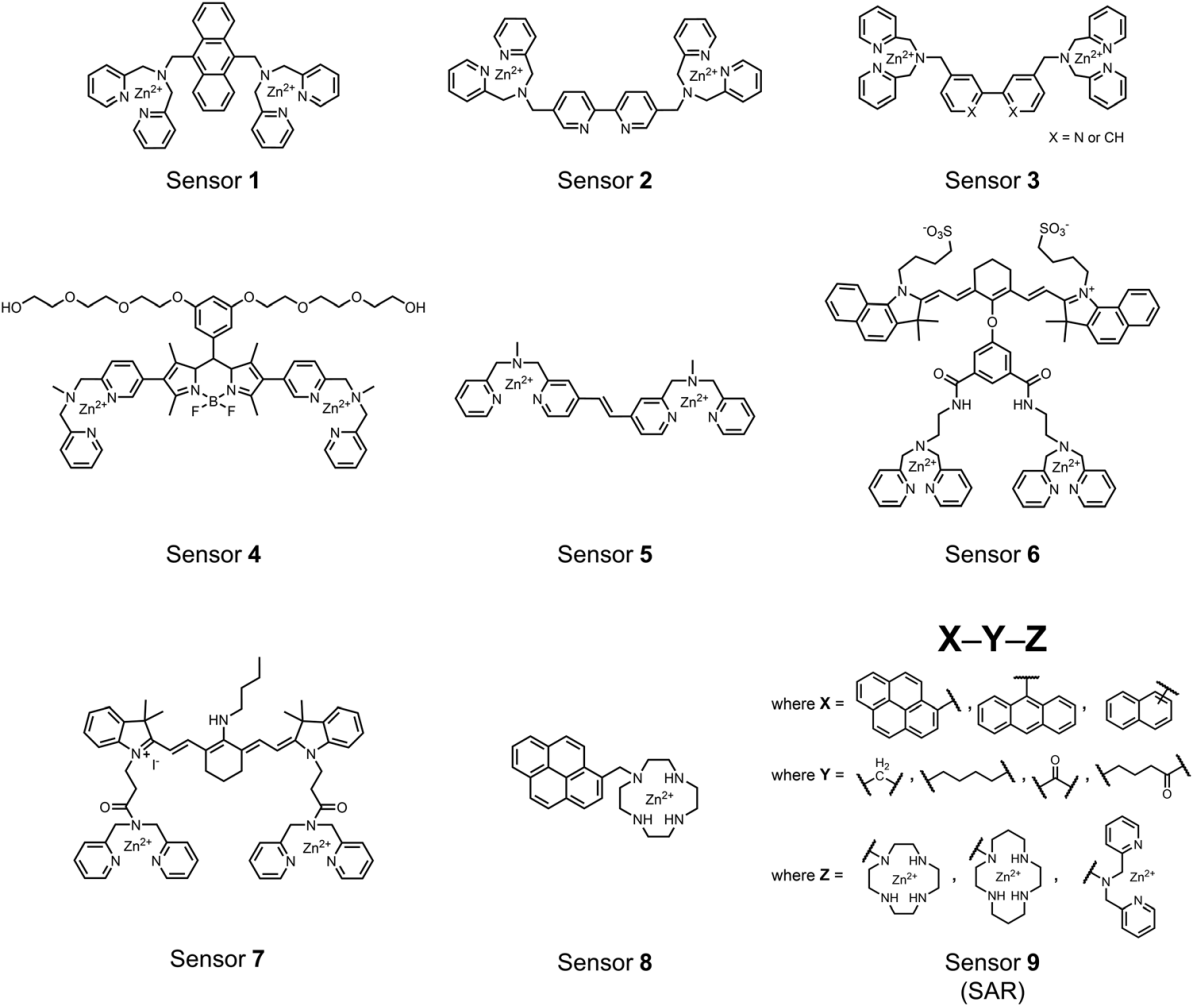
Chemical structure of fluorescent chemosensors **1–9**.

Small-molecule fluorescent chemosensors are the most explored detection method for proximal phosphorylation (Fig. 3). The chemosensor approach is prevalent due to simple preparation through chemical synthesis, inexpensive reagents, and compatibility with a variety of assays. The structural template for fluorescent chemosensors is commonly comprised of a fluorophore transducer, a phosphate-interaction motif (referred to as ‘receptor’), and a functionalized linker. The fluorophore is selected based on conventional probe parameters including excitation/emission wavelengths, quantum yield, photostability, and solubility. The phosphate-binding receptor typically employs cationic substituents or transition-metal centres to engage anionic phosphates through attractive charge interactions. Optimal linker chemistry is important for tuning distance requirements and flexibility to allow for detection of spatially proximal phosphates, as well as maintaining chemosensor solubility. Each of these chemical subunits can be adapted and modified to create a specialized chemosensor for a variety of substrates, environments, and detection methods.

A fluorescence-based small-molecule chemosensor approach for phosphorylated peptide detection is highlighted in the pioneering work by the Hamachi group. The chemosensor **1** utilized an anthracene fluorophore two Zn(ii)–dipicolylamine (DPA) receptors to detect mono-phosphorylated peptides in aqueous solution.^54^ The bidentate receptor employed Zn(ii) which, similar to enzymatic phosphate receptor pockets, achieved selective binding to phosphates through coordinative and electrostatic interactions. With this approach, chemosensor **1** achieved moderate selectivity over common anions (sulfonate, nitrate, acetate, chloride), and bound phosphate *via* a 1 : 1 binding interaction in fluorescence titrations (50 mM HEPES buffer, pH 7.2). The same chemosensor **1** was later shown to detect a proximally phosphorylated protein in aqueous solutions and on polyacrylamide gels.^55^ Herein, sensor **1** was shown to detect α-casein, a commonly employed model phosphoprotein harbouring 8–9 phospho-sites in two spatial clusters.^56^ The sensor was employed as an SDS-PAGE stain which visibly stained α-casein at a higher level than phosphorylated ovalbumin (2 distal phospho-sites).^57^ Although the sensing mechanism was not selective for proximal phosphates in this iteration, this initial chemosensor was able to detect levels as low as 500 ng α-casein by SDS-PAGE and 250 nM in fluorescence-based protein titrations (50 mM HEPES buffer, pH 7.2). Thus, the small-molecule fluorescent chemosensor approach was demonstrated to be a viable method for detecting phosphorylated peptides in physiological aqueous conditions.

Hamachi and coworkers sought to apply their sensing methodology to the selective detection of di-phosphorylated peptides. The bis(Zn(ii)–DPA) receptors were linked *via* a 2,2′-bipyridine at various positions to yield sensor **2**.^58^ The scaffold was designed to cross-link proximal phosphates by adjusting the spacing between the DPA receptors on the bipyridine spacer. This strategy was successful for proximal pS and pY motifs on model peptides.^58^ Circular dichroism (CD) and ^31^P NMR spectroscopy validated proximal detection as well as selection of the appropriate spacing for the bisDPA receptor subunits to engage with proximal phosphates. This optimal spacing was determined to be the 5,5′ positions, or *para* to the aryl–aryl bond of 2,2′-bipyridine. This rigid placement of the receptors afforded the highest fluorescence-fold change for a model peptide which had pS residues of *i*,*i* + 7 spacing (**pS**AKEAAA**pS**). Importantly, this distance was designed to approximately correspond to the Zn^2+^–Zn^2+^ chemosensor distance (11–13 Å) allowing for optimal binding geometry. The difference in binding for the *i*,*i* + 7 peptide over a *i*,*i* + 4 and *i*,*i* + 11 proximal pS peptide was relatively small (3-fold), which was attributed to the flexibility of the peptides in aqueous solution. However, this strategy could be limited to specific phosphate spacings on more complex peptides or proteins due to the inherent rigidity of the scaffold. Nonetheless, the sensor was able to detect down to 1 μM phosphopeptide and was selective over mono- and non-phosphorylated peptides (10 mM borate, pH 8.0, 50 mM NaCl). Thus sensor **2** illustrated that this class of chemosensors could distinguish between single and di-phosphorylated analytes.

The Hamachi group subsequently utilized their small-molecule chemosensor approach for the inhibition of protein–protein complex formation.^34^ Probes were synthesized with bis(Zn(ii)–DPA) receptors to interact with a proximal pSpS peptide moiety. This sequence corresponds to the C-terminal domain (CTD) of RNA polymerase II and is critical in complexation with the WW domain of the peptidyl–prolyl isomerase Pin1.^59^ To interrupt the CTD/WW domain complex, the authors appended the Zn(ii)–DPA binding unit to various aryl linkers at different positions. The most effective phosphate receptors to the CTD (*i*,*i* + 3 pSpS sequence) contained two Zn(ii)–DPA binding units on either a biphenyl or bipyridyl linker appended to the 4,4′ positions. Similar to sensor **2**, the 4,4′ positions afforded an optimal Zn^2+^–Zn^2+^ distance (11.5 Å) to the *i*,*i* + 3 pSpS sequence (9.7 Å). Binding to the CTD pSpS moiety was validated by ITC and CD spectroscopy. ^31^P NMR confirmed the interaction occurred *via* a bidentate coordination involving both Zn(ii)–DPA receptors. To evaluate the inhibitory activity, fluorescence anisotropy was performed with rhodamine-labeled CTD peptides (50 mM HEPES, pH 7.2). Fluorescence anisotropy determined that the biphenyl probe **3** was the most effective at competitively displacing CTD from the WW domain (*K*_d_ = 123 ± 6 nM). These results demonstrate that selective probes could be used for both the detection and inhibition of proximally phosphorylated peptide sequences.

Small-molecule fluorescent chemosensors have also been used for the detection of phosphorylated tau (pTau), a neuronal microtubule-associated protein implicated in several neurodegenerative diseases, including Alzheimer's disease. In Alzheimer's pathophysiology, tau is abnormally hyper-phosphorylated at more than 30 sites, leading to microtubule disassembly and insoluble aggregation as neurofibrillary tangles (NFTs).^60^ Notably, pTau is hyper-phosphorylated in proximal clusters on either sides of the microtubule-binding domain which is a prerequisite for NFT formation and other conformational changes.^61^ As such, fluorescent chemosensors have a strong potential in understanding disease biology as well as diagnosis and assaying potential therapeutics. Chemosensor **4** was designed for this purpose, utilizing bis(Zn(ii)–DPA) receptors appended to a BODIPY fluorophore.^62^ This scaffold aimed to directly improve the previous bipyridiyl chemosensors with superior fluorescence properties. Quantum yield was increased from bipyridyl (*Φ* < 0.1) to BODIPY (*Φ* = 0.31) while excitation and emission wavelengths were red-shifted (BODIPY *λ*_ex/em_ = 520/537 nm; 50 mM HEPES, pH 7.2). Chemosensor **4** was capable of detecting various tau-derived pSpS and pSpT peptides over non- and mono-phosphorylated counterparts. Similar to previous chemosensors, **4** exhibited a strong and specific preference towards *i*,*i* + 4 spaced phosphorylated peptides. The rigid scaffold prevented binding to tau-derived di-phosphorylated peptides of *i*,*i* + 2 and *i*,*i* + 6 phosphate spacing, which showed no fluorescence changes upon incubation. Nonetheless, chemosensor **4** was successful in detecting tau phosphorylated by GSK-3β with an EC_50_ of 9 nM. Binding to pTau aggregates was presumed to be both due to Zn(ii)–phosphate interactions and favourable hydrophobic packing of the BODIPY core to the protein backbone. Chemosensor **4** was also successful as a stain for fluorescence microscopy of pTau aggregates. Histological imaging of NFTs in an Alzheimer's patient brain tissue sample showed colocalization of the BODIPY chemosensor with a monoclonal antibody for pTau. These experiments validated the usage of small molecule chemosensors for detection of (proximally) hyperphosphorylated protein targets. Furthermore, chemosensor **4** was also employed in the real-time fluorescence detection of GSK3β-catalyzed phosphorylation.^63^ Remarkably, the sensor could fluorescently monitor the proximal phosphorylation reaction of GSK3β with the substrate peptide amidst the presence of ATP and ADP. As a proof-of-principle, increasing concentrations of a known inhibitor for GSK3β were added into a mixture with substrate peptide, and the corresponding reduced fluorescence enhancement tracked with increasing inhibitor concentration. The resulting inhibition constant (*K*_i_ = 45 nM) was consistent with the reported value for the inhibitor, demonstrating the applicability of the chemosensing system for screening GSK3β inhibition. Chemosensor **4** has shown that the utility of chemosensors extends to active biological systems like enzymatic reactions, pTau aggregates, and patient tissue samples.

Expanding upon the previous bipyridiyl chemosensors, the Hamachi group sought to improve sequence selectivity for *i*,*i* + 1 diphosphopeptides. To accomplish this, chemosensor **5** was designed with bis(Zn(ii)–DPA) conjugated within a *trans*-4,4′-diazastilbene fluorophore.^64^ Conjugation of the phosphate–receptor and fluorescence reporter led to a red-shifted emission change upon Zn(ii)–phosphate binding from 387 nm to 427 nm. Although the quantum yield was low (*Φ* < 0.01), the red-shifted emission allowed for ratiometric sensing (427 nm/387 nm) which increased sensitivity of the system. The ratiometric signal allowed for detection down to 2 μM diphosphopeptide in aqueous solution (50 mM HEPES, pH 7.2). The rigidity of the diazastilbene core provided sequence selectivity for *i*,*i* + 1 diphosphopeptide over further spaced phosphates (*i*,*i* + 2, *i*,*i* + 3, *i*,*i* + 4) as well as inorganic phosphate.

Chemosensors for detecting pTau aggregates have also been improved. In physiological conditions, tau is intrinsically disordered but hyperphosphorylation induces their aggregation to NFTs as ordered β-sheets.^65^ Due to the stacking of β-sheets in single molecule layers,^66^ the proximal clusters of monomeric tau proteins are in the same position and proximity as aggregates, allowing for NFT detection *via* proximal phosphorylation sensing. This rationale was proposed in the design strategy of a ratiometric fluorescent chemosensor for pTau and NFT detection.^67^ The chemosensor **6** contained the NIR cyanine dye indocyanine green as the backbone of the sensing scaffold due to its beneficial optical properties for *in vivo* applications. Zn(ii)–DPA receptors were appended to the backbone, where a bisDPA receptor yielded the most potent fluorescence response (pTau EC_50_ = 0.27 μg mL^−1^) as compared to a mononuclear DPA receptor (pTau EC_50_ = 1.23 μg mL^−1^; 5 μM sensor, 50 mM HEPES, pH 7.4, 10% DMSO). Potent detection was observed with a ratiometric absorption mechanism (810/750 nm), which increased in the presence of pTau from as little as 100 ng, and consequently decreased with non-phosphorylated Tau. *Ex vivo* pTau was also detected from AD human and mouse brains (P301L) samples *via* fluorescence-based sample titration down to 10 μg mL^−1^. Fluorescence microscopy imaging identified NFT aggregates within brain extract samples by treatment of chemosensor **6** (10 μM, *λ*_ex_/*λ*_em_ = 783/800 nm). The fluorescence signal was abolished by the addition of PPi (100 μM), which further demonstrated the phosphate-specific mechanism of binding. SDS-PAGE staining was also applied with chemosensor **6**, and was co-validated by the total phosphorylation stain Pro-Q Diamond (10 μM sensor, 50 mM HEPES, pH 7.4, 10% DMSO). Therefore, the NIR chemosensor **6** was able to detect AD-derived pTau and NFTs through a ratiometric and phosphate-dependent approach.

In addition to direct fluorescence emission-based sensing, Ge and Tian reported the Cy7-based bis(Zn(ii)–DPA) sensor **7** for fluorescence lifetime imaging of pTau.^68^ The probe was designed with bis(Zn(ii)–DPA) receptors to bind *i*,*i* + 4 proximal phosphates, as well as a cyanine fluorophore with a strong Stokes shift (*λ*_ex/em_ = 600/760 nm). Fluorescence lifetime decays were measured for sensor **7***in vitro* in response to pTau, achieving detection down to 85 nM while being selective over other bioanalytes including amyloid-β, non-phosphorylated tau and ATP (10 μM probe, pH 7.4, cell lysis buffer). A PET mechanism induced by fluorescence quenching of bound Zn(ii) facilitated binding by increasing fluorescence lifetime when bound to pTau. The PET mechanism was demonstrated by computational and experimental quantum yield measurements, where unbound probe fluorescence (*Φ* = 0.027) increases significantly upon binding (*Φ* = 0.281, 9 μM pTau). For *in cellulo* experiments, fluorescence lifetime imaging microscopy (FLIM) was used to detect pTau in primary cultures of mouse cortical neurons. Sensor **7** was able to detect pTau in single neurons and could detect higher amounts of pTau when oxidative stress was applied (100 μM H_2_O_2_, 6 days). Thus, an improved cyanine reporter allowed for increased applications of the bis(Zn(ii)–DPA) receptor for proximally phosphorylated tau.

Up to this point, small-molecule fluorescent chemosensors were selective for specific spacings (*i*,*i* + *n*) of proximal phosphorylation motifs on peptide sequences. This selectivity was due to rigid receptor scaffolds which locked Zn(ii) atoms at specific distances allowing for preferential binding of diphosphate residues positioned at the complementary distance. Moreover, chemosensors were designed to form a 1 : 1 complex with the proximal phosphate motif, whereby one sensor molecule simultaneously engages both proximal phosphates.

The Gunning group sought to develop a small-molecule fluorescent chemosensor for proximal phosphorylation motifs of variably spaced di-phosphate motifs. To accomplish this, an excimer reporting mechanism with a pyrene fluorophore was employed.^69^ Chemosensor **8** was designed as a mononuclear receptor of Zn(ii)–cyclen bound to a pyrene fluorophore through a short, flexible methylene linker. Compared to previous small-molecule chemosensors, the mononuclear receptor would bind to only one phosphate moiety. However, when these phosphates (and multiple bound-chemosensors) were in close proximity, the pyrene fluorophores could stack through π–π interactions and form an excited-dimer or excimer with a red-shifted emission (*λ*_ex/em_ = 350/476 nm). This excimer signal was consistent with a 2 : 1 sensor-motif binding that was found to be cooperative (*n*_Hill_ = 3.2 ± 0.2) and allowed detection of a ApYpYAA peptide down to 625 nM in fluorescence-based peptide titration (50 mM HEPES, pH 7.5, 10% DMSO). Common phosphoproteins including α-casein and β-casein were able to be detected down to 80 nM in titration experiments (50 mM HEPES, pH 7.5, 20% DMSO, 75 mM NaCl). On polyacrylamide gels, sensor **8** detected as little as 1.2 μg protein and could differentiate between proximal (β-casein) and distally phosphorylated (STAT5) proteins as a gel stain (50 mM NaOAc, pH 5.5, 5% DMSO, 25 mM NaCl, 1 h staining). The Gunning group demonstrated that the unique excimer mechanism allowed chemosensor **8** to detect proximal phosphorylation motifs of variable spacing, on both peptides and proteins.

Chemosensor **8** was employed alongside a sequestering receptor to improve sensitivity for proximal phosphates over common physiological off-targets, including ATP and pyrophosphate (PPi).^70^ The sequestering receptor was designed as a triethylbenzene scaffold functionalized with three indoles, where two of the three indoles were appended with Zn(ii)–cyclen receptors. The cavity induced by the indoles would allow for the binding of low molecular weight phosphoanions such as ATP and PPi but prevent the binding of larger phosphopeptides or proteins. This sequestering phosphate receptor was able to improve the selectivity for sensor **8** signaling for a A**pYpY**AA (29% signal-to-noise reduction) over PPi (75% signal-to-noise reduction) by ∼3 fold. This demonstrated the applicability of ‘sequestering phosphate receptors’ for improving proximal phosphorylation selectivity and sensitivity. In further studies, characterization of the excimer fluorescence assays with chemosensor **8** were investigated, including buffer, metal compatibility, and peptide phosphate motifs.^71^ In these characterization studies, it was determined that sensor **8** was able to detect selectively proximal phosphopeptide motifs of various residues (pY, pS, pT) and spacings (*i*,*i* + 1 − *i*,*i* + 6) over non-phosphorylated counterparts. Other proximally phosphorylated proteins phosvitin and riboflavin binding-protein were also detected. The sensitivity of the receptor was evaluated with the use of other transition metals (such as Fe^2+^, Cu^2+^, Mn^2+^, Ni^2+^) although only Zn^2+^ elicited a response to a A**pYpY**AA peptide. Sensor **8** was also applied to detecting the loss of proximal phosphorylation of model peptides and proteins *via* the activity of a phosphatase enzyme in the presence of Pi. The combination of chemosensor **8** alongside a sequestering receptor allows for selectivity between analytes which facilitates the analysis of more complex biological environments.

Further improvements on the chemosensor **8** scaffold were identified through a structure–activity relationship (SAR) study.^72^ The sensor structure (**9**) was modified at the three components: fluorophore, receptor, and linker. Initially, polybenzene aromatic fluorophores were evaluated for excimer capabilities, but neither anthracene nor naphthalene were able to elicit suitable excimer responses. The receptors assessed included cyclen, cyclam and DPA, which revealed differences in selectivity. Notably, a PET mechanism was evidenced for DPA-containing sensors by fluorescence spectral measurements, while cyclen and cyclam-based sensors were strictly ratiometric *via* a monomer–excimer equilibrium. Ideal linkers were evaluated with either 1, 2, or 4 methylenes, with either alkyl or amide linked to the receptor. Interestingly, rigidity in this scaffold *via* amide linkers abolished all fluorescence for proximally phosphorylated peptides, which contrasted with previous bis(Zn(ii)–DPA) sensors. Extending the length of linkers did not affect selectivity in pYpY peptide experiments, which was hypothesized to be due to the inherent flexibility of both the peptides and sensors in aqueous solution. Sensors were unable to distinguish between proximally phosphorylated peptides and mono-phosphorylated peptides with multiple carboxylate-containing amino acids. Furthermore, sensors were limited by the detection of nucleotide di/triphosphates and inorganic phosphate but could be used to monitor the progress of ATP hydrolysis reactions. Finally, SDS-PAGE of cell lysates revealed increased specific staining of presumably proximally phosphorylated proteins over the less selective stains Pro-Q diamond (total phosphorylation) and SYPRO Ruby (total protein) with detection limits >500 ng of multi-phosphorylated protein (β-casein). This SAR evaluated chemosensor **8** with both PET and ratiometric detection and showed promising utility as a selective SDS-PAGE stain.

Small-molecule chemosensors have been successful in detecting proximal phosphorylation motifs. To date, all chemosensors have incorporated chelated Zn(ii), which efficiently interacts with phosphate residues through electrostatic and/or coordinative interactions. Sensor scaffold rigidity has been shown to play a major role in proximal phosphate sequence selectivity, where bidentate receptor scaffolds require the inter Zn^2+^–Zn^2+^ distance to match the substrate diphosphate distance. Improvements towards sensor sensitivity have been shown by incorporating ratiometric mechanisms, whereby peptides and proteins have been detected down to low nanomolar concentrations. A trend toward more red-shifted reporters have improved optical fluorescence properties. Both 1 : 1 and 2 : 1 sensor-motif mechanisms have been shown to be effective for detecting proximal phosphates. Chemosensors have been applied to a variety of fluorescence-based assay formats, including fluorimetry, microscopy, and gel staining. The ability to rationally design scaffolds and facile synthesis for small-molecule chemosensors continues to be major driving forces behind the advancements in this field.

## 3. Luminescent chemosensors

Lanthanides are valuable optical reporters due to their unique luminescent properties that arise from the 4f electron shell. Luminescence occurs from spin ‘forbidden’ f–f orbital transitions by Laporte parity rules which causes extended excited state lifetimes (milliseconds) as compared to the fluorescence timescale (nanoseconds) enabling time-resolved measurements.^73,74^ Although this eliminates significant background noise from scattered light and autofluorescence, the forbidden f–f transitions result in weak absorption and small extinction coefficients.^73,74^ Lanthanides can be excited or ‘sensitized’ indirectly through transfer of excitation energy from a nearby organic fluorophore ‘antenna’.^73,74^ These antennas are responsive to environmental changes, allowing for their usage in chromophore-sensing mechanisms.^73,75^ In the context of phosphate detection, lanthanides are useful components of binding receptors since they typically exist as trivalent cations in the +3 oxidation state and may form complexes of high coordination numbers.^75^ These properties allow lanthanides to be used for unique time-resolved experiments with a high signal-to-noise ratio and facilitate synergistic sensing with organic fluorophore antenna.

**Fig. 4:**
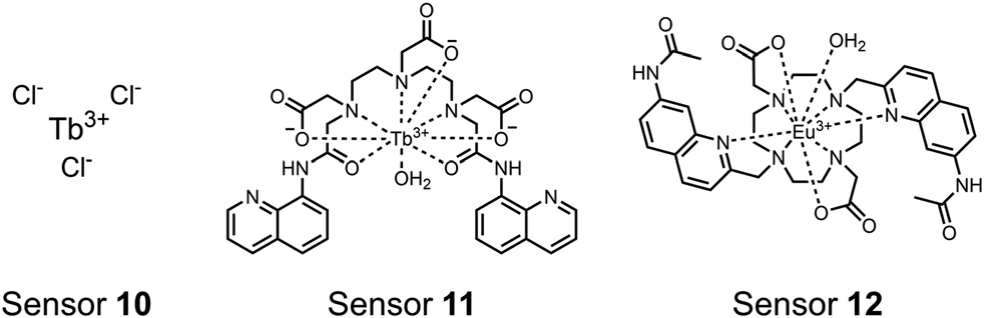
Chemical structure of lanthanide chemosensors **10–12**.

Lanthanides have been used to detect proximal phosphorylation motifs in a few interesting sensor designs (Fig. 4). A lanthanide metal centre can act as both the receptor, due to its cationic and coordinative interactions with phosphates, as well as the luminescent reporter. Lanthanides are commonly bound by chelating ligands but must have a vacant coordination site for engaging with phosphate moieties. When acting as a reporter, the requirement of an antenna for sensitization limits lanthanide sensor structures and/or analytes to those that contain appropriate aromatic groups.

Lanthanide sensors incorporating Tb(iii) have been shown to selectively detect mono-phosphorylated tyrosine-containing peptides.^76–78^ To apply this approach for proximal phosphorylation, the Gunning group developed an antenna-free Tb(iii) sensor **10** for detecting tyrosine-containing proximal phosphorylation motifs.^79^ The usage of TbCl_3_ salt in physiological aqueous solutions (50 mM HEPES, pH 7.5, 50 mM NaCl) allowed for the solvation of free Tb(iii), which acts as a Lewis acid for phosphate recognition. Once bound, a nearby aromatic group is necessary for sensitization to cause Tb(iii) luminescence. In a phosphotyrosine-containing proximal motif, the tyrosine aryl ring can act as the antenna for sensitization. Additionally, due to the lack of a chelator, the free Tb(iii) forms a more stable complex with proximally di-phosphorylated sites over mono-phosphorylated ones due to neighbouring group interactions. Therefore, the antenna-free TbCl_3_ sensor **10** was able to detect both a proximal pYpY and pTpY peptide in luminescence-based peptide titrations down to 4 μM. Di-phosphorylated peptides lacking a proximal pY residue or peptides with less than two phosphates resulted in low luminescence signals. Tb(iii) luminescence detection of pY-containing di-phosphorylated peptides was also retained in the presence of full-length proteins at high TbCl_3_ concentrations. Thus, this facile approach demonstrates the applicability of lanthanide luminescence in specific proximal phosphorylation applications.

In another report, a chelated Tb(iii) sensor **11** was able to selectively detect proximal phosphoserine motifs.^80^ Tb(iii) was chelated by diethylenetriaminepentaacetate, which was appended with two 8-aminoquinoline (8-AQ) groups. Tb(iii) luminescence was selectively induced by tau-derived peptides containing proximal pSpS motifs over those containing single pS or non-phosphorylated residues (25 mM sensor, 50 mM Zn(ii), 5 mM Tris HCl, 50 mM NaCl, pH 7.4, *λ*_ex/em_ = 250/545 nm). In contrast to the previous sensor, this design integrated the 8-AQ antenna for sensitization. When unbound, the 8-AQ antenna was PET quenched by a nearby secondary amine, preventing energy transfer to Tb(iii). However, in the presence of two auxiliary Zn(ii) centres and a di-phosphorylated peptide, Zn(ii) coordination between 8-AQ and the phosphate residues occupies the secondary amine lone pair, which deactivates PET quenching and allows for sensitization and Tb(iii) luminescence. With this mechanism, sensor **11** could detect >62 nM pSpS peptide *via* time-resolved luminescence utilizing a short delay (50 μs). Proximal phosphoserine motif sensing was most enhanced by those of closest proximity (*i*,*i* + 1 > *i*,*i* + 2 > *i*,*i* + 3), likely due to the tight spatial constraints of the chelate structure. Sensor **11** was selective against common anions and nucleotide phosphates, although PPi potently induced luminescence signal. Finally, sensor **11** could detect exogenously introduced pSpS peptide into a brain homogenate sample *via* the time-resolved measurements. Sensor **11** illustrated the selectivity between peptides you can achieve by using a Tb(iii) chelate and showed the viability of this technique in patient samples.

Apart from Tb(iii), the lanthanide Eu(iii) has also been used in chemosensors for luminescent detection of phosphotyrosine-containing peptides.^81,82^ To achieve sequence selectivity for pYpY proximal peptides, Hewitt and coworkers utilized the modified-DOTA chelated Eu(iii) complex sensor **12**.^83^ The octadentate chelate was a DOTA-derived ligand modified with two amidated quinoline groups that were previously proposed to strengthen binding to phosphates *via* hydrogen bonding.^84^ Selective luminescence enhancement was observed by sensor **12** and a pYpY peptide over YY and YpY motifs through a ratiometric signal of 612/599 nm (8 μM sensor, 10 mM HEPES, pH 7.0, *λ*_ex_ = 330 nm). Signal was minimal for non-phosphorylated and mono-phosphorylated tyrosine motifs as well as proximal pSpS and pTpT peptides. Notably, only minor enhancement was observed for distal *i*,*i* + 2 and *i*,*i* + 3 pYpY peptides. This sequence selectivity was proposed to be a spatial requirement of both phosphate groups binding to the Eu(iii) centre, displacing the bound water molecule and possibly one quinoline group, favouring enhancement with the closer *i*,*i* + 1 pYpY motif. Sensor **12** was applied to monitor phosphatase-catalysed dephosphorylation (8 mM peptide, 0.5 units per mL acid phosphatase, 10 mM HEPES, pH 7.0). Decreasing luminescence was monitored in real-time and the reaction rate was observed to increase upon the addition of higher concentrations of phosphatase enzyme. Sensor **12** expanded lanthanide metal diversity with Eu(iii) and demonstrated its usage in monitoring proximal phosphorylation enzymatic reactions.

Luminescent sensors have been effective in detecting proximal phosphorylation motifs on peptides. Both Tb(iii) and Eu(iii) lanthanide metal centres have been employed for their strong spectroscopic properties. Lanthanides have been used in chelate complexes or as ligand-free sensors. These sensors have used multiple reporting mechanisms including time-resolved luminescence and sensitizer-PET quenching. A variety of phosphate recognition mechanisms have been demonstrated, including direct lanthanide metal binding, chelate-only binding, and dual lanthanide-chelate binding. The current designs of luminescence sensors have demonstrated sequence selectivity for adjacent proximally phosphorylated residues due to spatial constraints in the chelate complex. Finally, the strong luminescent properties of time-resolution, large Stokes shifts from sensitizer to lanthanide, and narrow emission bands have allowed for real-time measurements and detection within complex biological environments. These unique characteristics can allow for considerable advancements in the applications of sensors for proximal phosphorylation.

## 4. Hybrid biosensors

Biosensors define a wide range of molecular recognition scaffolds that incorporate biological components in the sensor design. The addition of a biological element is employed for analyte specificity or to access complex sensing modalities not available to small-molecule chemosensors, such as enzyme-catalyzed sensing.^85^ The biological element can vary in size from small polypeptide chains, DNA strands, and protein domains, to larger enzymes, antibodies, organelles and whole-cells.^85^ Within the types of signal transducers for biosensors, optical reporters are the most common.^86^ The optical reporter can be fitted to the biological structure through chemical, enzymatic or genetically-encoded means.^85,87,88^ The ability to construct and modify biological components through molecular biology and protein engineering has allowed fine-tuning of recognition processes and sensing of a wide variety of biorecognition events.^88,89^ Biosensors can utilize unique sensing mechanisms through large conformational changes that are unavailable to small-molecule chemosensors. For example, intramolecular FRET (Förster resonance energy transfer) requires a significant spatial distance between donor and acceptor chromophores (1–10 nm) typically achieved through conformational changes of the biological component.^90^ Biosensors can be exceedingly analyte-specific with recognition elements such as nucleic acids for strand complementarity and antibodies for antigen agglutination.^85,89^ Thus, biosensors are effective analytical tools that have the potential to create innovative solutions for several research fields.

**Fig. 5:**
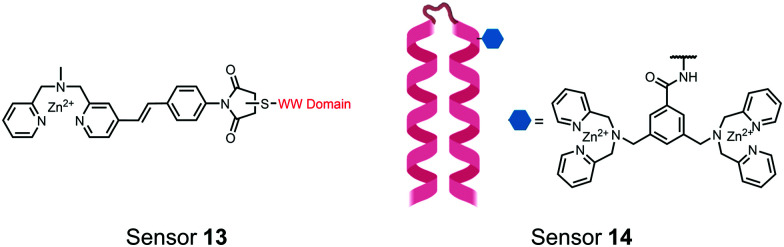
Structures of hybrid biosensors **13** and **14**.

The primary impact of biosensors on proximal phosphorylation detection has been the mixing of biological and chemical components to create hybrid biosensors tailored to cooperative binding of specific analytes.^91,92^ The addition of the chemosensory component to a biological receptor can allow for increased binding affinities and detection of previously intractable targets.^91,92^ Although more complex biological components such as antibodies, engineered proteins and aptamers are commonly used for potent binding, these technologies are often costly, arduous, and complex to make. A more facile and robust approach uses smaller biological components such as polypeptides or specific protein domains, as seen in the following proximal phosphorylation sensor examples (Fig. 5).

The Hamachi group explored biosensors for proximal phosphorylation by combining a small-molecule chemosensing receptor with a protein domain to create a hybrid biosensor.^93^ They utilized the Pin1 WW domain which preferentially binds phosphorylated proline-rich sequences (pS-Pro and pT-Pro) such as on the multiphosphorylated C-terminal domain of RNA polymerase II.^94^ In a previous study, X-ray crystallography revealed that the WW domain binds to only one pS residue in a doubly phosphorylated peptide (Y**pS**PT**pS**PS).^95^ The authors hypothesized that by adding a Zn(ii) binding motif to the WW domain, more potent and selective binding could be achieved for the pSpS peptide. Therefore, sensor **13** was designed with a Zn(ii)–DPA receptor derived from a stilbazole core, which was appended to the WW domain *via* a maleimide handle and a mutated cysteine residue. CD spectroscopy confirmed that cysteine mutation and chemosensor affixture still resulted in an appropriately folded WW domain. The stilbazole moiety was selected due to its propensity for fluorescence intensity changes upon conformational restriction. When unbound, the aromatic rings retain some rotational freedom, but upon binding the constrained sterics resulted in changes to the fluorescence emission profile. As postulated, fluorescence emission increased upon the introduction of di-phosphorylated peptide down to 1 μM (*λ*_ex/em_ = 340 nM/440 nm, 50 mM HEPES, pH 7.2). Fluorescence polarization also confirmed the binding mechanism, where increased anisotropy related to loss of fluorophore mobility. Cooperative activity of both chemosensor and biosensor components was validated by the calculated *K*_app_ of 1.2 × 10^6^ M^−1^, which was *ca.* 10-fold larger than the native WW domain and *ca.* 1000-fold larger than the mono-nuclear Zn^2+^ complex. Sequence selectivity was demonstrated over a pY containing peptide (**pY**ETD**pY**), where no difference was observed between mono-phosphorylated or di-phosphorylated forms due to WW domain motif specificity. Sensor **13** was applied to real-time detection of a kinase-catalyzed peptide phosphorylation reaction. A mono-phosphorylated **pS**PTS peptide was monitored for a second phosphorylation event on serine by cyclin-dependent protein kinase 9 (CDK9). In this assay, sensor **13** ignored all other interferants within the reaction mixture and was able to determine the Michaelis–Menten kinetics for the CDK9 phosphorylation event.

In another strategy, α-helical polypeptides were conjugated to a bis(Zn(ii)–DPA) moiety to detect proximally phosphorylated proteins.^96^ The hybrid biosensor **14** was constructed with polypeptides of 42-amino acids that formed two amphiphilic helices connected by a short loop. The chemical binder was appended to a lysine residue to combine coordinative and electrostatic Zn(ii)–phosphate interactions with polypeptide backbone hydrophobic packing for detecting target proteins. CD spectroscopy confirmed the retention of the helical secondary structure. To ascertain biosensor affinity for proteins, the fluorophores coumarin or fluorescein were appended to an orthogonal lysine residue. Fluorophore quantum yield was altered due to environmental changes upon engagement with target proteins. Selectivity was demonstrated for proximally phosphorylated α-casein and β-casein over mono-phosphorylated ovalbumin (500 nM sensor, 10 mM HEPES buffer, pH 7.2, 150 mM NaCl). Among polypeptide sequences tested, the best binders had a positive net-charge of +2. In a control experiment, Zn(ii)-free biosensor **14** prepared with or without the bisDPA moiety showed no response to proteins, demonstrating that additional interactions afforded by Zn(ii) are necessary. In another application, biosensor **14** was functionalized through a loop Cys disulfide bond to coated beads for pull-down experiments. After α-casein treatment and washing, biosensor **14** was released by the addition of DTT and the captured proteins were analyzed by SDS-PAGE. This assay was able to capture α-casein (>10 nM) and enrichment was not inhibited by 10 mM phosphate buffer or 400 μM of a mono-pY peptide in the presence of <500 nM α-casein. Thus, the hybrid biosensor **14** demonstrated versatility as an optical sensor and pull-down enrichment tool for proximally phosphorylated proteins.

The sensing of protein phosphorylation has been achieved through a variety of biosensors, although this remains an heavily underexplored area.^2,97^ The above reports demonstrate the advantages of a hybrid biosensor for detecting proximal phosphorylation motifs including sequence selectivity and improved binding potency through the cooperative interactions of a chemical and biological component. The chemosensor component can aid sensing beyond being only an optical reporter such as a fluorescent dye or chromophore. The reports have shown that the artificial component can be tailored as a co-binder for improved binding efficiency. The specificity in detecting specific proximal phosphorylation motifs demonstrates the strengths of this modality and the opportunities within the field for future hybrid development.

## 5. Inorganic nanoparticles

**Fig. 6:**
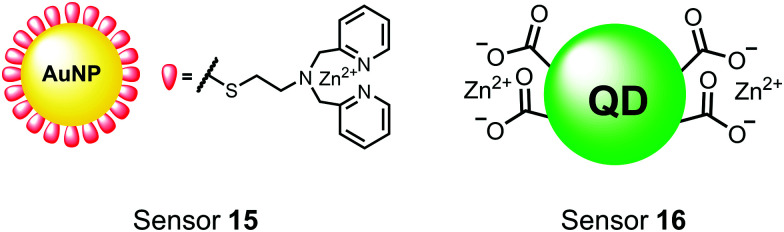
Structures of inorganic nanoparticle sensors **15** and **16**.

Nanoparticles have drawn substantial interest for optical sensing due to unique photophysical properties that derive from their nano-scale dimensions. These nanomaterials exist on the scale of 1–100 nm and exhibit a diverse range of physical and chemical properties that differ from single-molecules or bulk materials of the same composition.^98,99^ Their characteristics include a large surface-to-volume ratio, high reactivity and optical readouts arising from surface plasmon resonance that depend on their size, shape, and aggregation.^98^ Gold is the most commonly explored metallic nanoparticle for a variety of applications including sensing, therapeutics and drug delivery.^99,100^ Gold nanoparticles (AuNPs) are routinely used in biological applications because of their inert stability, well-developed synthetic methodologies and modulatable surfaces.^99,100^ For sensing applications, AuNPs can be chemically functionalized with target-specific receptors for specific binding of analytes in biological applications.^99^ Another important class of nanoparticles for optical sensing are quantum dots, which are semiconductor nanocrystals that emit photoluminescence with discrete, tunable wavelengths.^101,102^ Due to their small size (nm), quantum confinement effects arise where the electron bandgap and resulting emission can be tuned based on particle size and chemical composition.^101^ When compared to traditional fluorescent dyes, quantum dots have superior optical properties including high quantum yields, long luminescence lifetimes, narrow emission bands and stability towards photobleaching.^101,102^ The excellent qualities of quantum dots have expanded optical sensing applications where organic fluorophores are inadequate.^102^ The following reports describe inorganic nanoparticles used for proximal phosphorylation applications (Fig. 6).

Zhang and colleagues developed a AuNP outfitted with Zn(ii)–DPA for the detection of di-phosphorylated peptides.^103^ They chose to outfit sensor **15** with Zn(ii)–DPA due to its success as a receptor in small-molecule chemosensors popularized by the Hamachi group. The authors proposed that when sensor **15** was mixed with di-phosphorylated peptides, aggregation of the AuNPs would cause a colorimetric change. The authors selected the proximally phosphorylated peptide Y**pS**PT**pS**PS derived from the C-terminal domain of the RNA polymerase II large subunit as the analyte of interest. Upon addition of 121 μM peptide, sensor **15** underwent a visual colorimetric change from red to purple (5 nM AuNPs, DI water). UV-vis spectra showed an absorption peak at 521 nm decreasing while a band at 680 nm appeared and was gradually red-shifted upon higher concentrations of peptide. This colorimetric change supported the AuNP aggregation mechanism and was further validated by TEM images that showed colloid formation. Other proximally phosphorylated pSpS peptide sequences (GG**pS**GG**pS**G, GG**pS**G**pS**GG) also induced a colorimetric change while non-phosphorylated variants and mono-phosphorylated controls did not. The color change was attributed to a cross-linking effect, where several bound AuNPs form large colloids that cause a red-shifted absorbance *via* surface plasmon resonance absorption.^99^ Notably, the AuNP-based sensor was sensitive to ionic strength, where a buffer of 50 mM HEPES, pH 7.2 induced agglomeration of the colloid without analyte present. Improvements to the stability of these AuNPs would be necessary to use the chemosensor in more complex media. Nonetheless, sensor **15** demonstrated the ability for an outfitted AuNP to selectively detect proximally di-phosphorylated peptides over non- and mono-phosphorylated ones *via* a colorimetric mechanism.

In another report, Lim and coworkers outfitted quantum dots with Zn(ii)-coordinating ligands to construct an *in vitro* screening tool for protein kinase activity by FRET.^104^ The quantum dot sensor **16** was used as the FRET donors while target phosphopeptides were fluorescently-labelled with TAMRA to act as the FRET acceptor. Sensor **16** was outfitted with carboxylate residues to coordinate transition metals for phosphate binding. Among metal ions employed (Ni^2+^, Co^2+^, Cu^2+^, Fe^2+^), only Zn^2+^ resulted in FRET signal when sensor **16** was mixed with labelled phosphorylated peptides (2 nM sensor, 100 μM Zn(ii), 20 mM Tris–HCl buffer, pH 7.4, *λ*_ex_ = 380 nm, *λ*_em_ = 450–650 nm). **16** was able to detect >80 nM of mono-pS peptide, while no FRET emission was observed with the non-phosphorylated variant. Another proximally di-phosphorylated peptide sequence **pS**PPQ**pS** derived from heat shock factor 1 protein also demonstrated a high FRET ratio. Among the same peptide sequence, a trend of di- > mono- > non-phosphorylated peptide detection was observed, demonstrating that higher amounts of phosphorylation improved FRET efficiency. However, in screening kinase reactions, the required high concentrations of ATP (160 μM) and Mg^2+^ (10 mM) interfered with the FRET enhancement of sensor **16**. To circumvent this, a streptavidin-coated microbead affinity purification of the peptide substrate was performed. This allowed for centrifugation and removal of the supernatant and isolation of peptide-bound microbeads. This allowed for signal enhancement of the FRET signal upon sensor **16** and Zn(ii) addition. Thus, the quantum dot chemosensor **16** was able to selectively detect increasingly phosphorylated peptides in an *in vitro* FRET assay.

Currently, sensors based on inorganic nanoparticles have been underutilized for the selective detection of proximal phosphorylation. From the existing work, the unique optical properties of nanoparticles have been used to detect proximal phosphosites *via* visual colorimetric detection and FRET. Although the optical properties of nanoparticles are attractive, the challenge of differentiating between biological anions, mono-phosphorylated residues, and proximally phosphorylated sequences is evident. Surface immobilization of several metal chelates for phosphate recognition are the current methods of proximal phosphorylation detection by nanoparticles, but provide limited selectivity over anionic off-targets. The ability to incorporate molecular receptors of correct spacing and length for specific proximal motifs could be more difficult for nanoscale structures as compared to small-molecule sensors. However, there is still great opportunity to employ nanoparticles for the detection of proximal phosphorylation. Large classes of inorganic particles such as carbon dots or magnetic nanoparticles have yet to be reported. Pairing with the correct receptor or detection through a dual-response of the nanoparticle surface could afford new selectivity towards proximally phosphorylated sequences (Table 1).

**Table 1:** Methods for the detection of proximal phosphorylation

Sensor	Structure	Target	Assay type	Buffer	LOD*	Ref.
Fluorescent chemosensors						
**1**	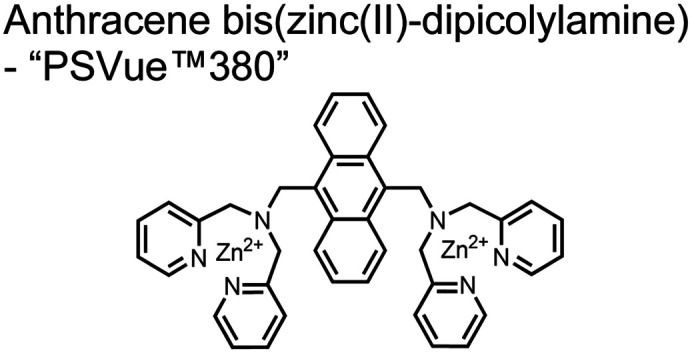	Mono-phosphorylated peptides, α-casein	SDS-PA gel staining, fluorescence spectroscopy	50 mM HEPES buffer, pH 7.2	500 ng α-casein *via* PA gel, 150 nM *via* fluorescence spectroscopy	*J. Am. Chem. Soc.*, 2002, **124**, 6256–6258
						*Chem. Lett.*, 2004, **33**, 1024–1025
**2**	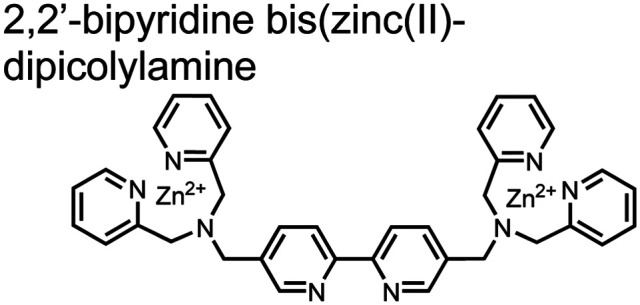	Various peptides containing proximal pS and pY motifs	Fluorescence spectra/titrations CD, ^31^P NMR	10 mM borate pH 8.0, 50 mM NaCl	1 μM phosphopeptide	*J. Am. Chem. Soc.*, 2003, **125**, 10184–10185
**3**	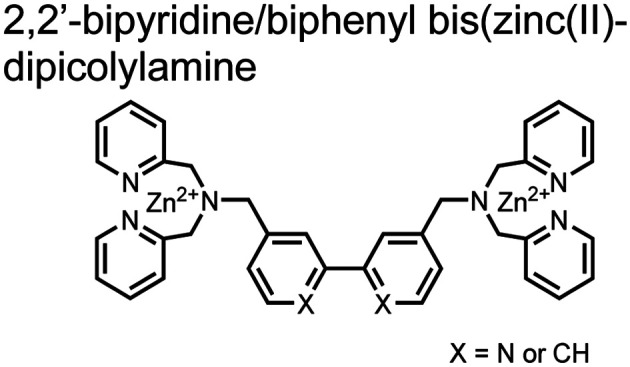	Proximal pSpS phosphopeptide	ITC, CD, NMR, fluorescence anisotropy disruption of CTD phosphopeptide/Pin1 WW domain	50 mM HEPES, pH 7.2; 10 mM borate pH 8.0	2.5 μM phosphopeptide	*J. Am. Chem. Soc.*, 2006, **128**, 2052–2058
**4**	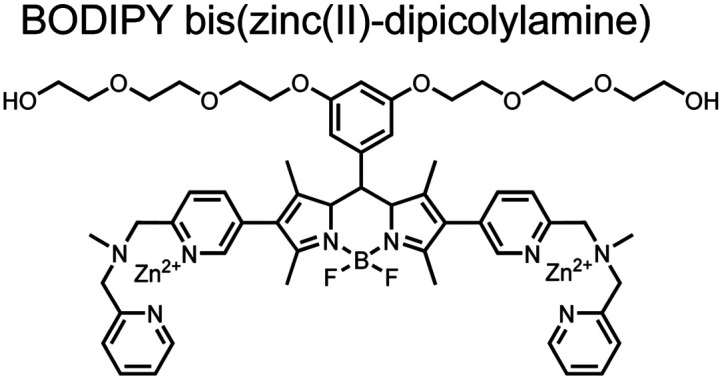	Proximally phosphorylated pTpS Tau peptides	Fluorescence titration, microscopy, immunological staining; GSK3β-catalyzed reaction tracking	50 mM HEPES, pH 7.5	2 μM peptide in titrations	*J. Am. Chem. Soc.*, 2009, **131**, 6543–6548
						*Bioorg. Med. Chem. Lett.*, 2009, **19**, 4175–4177
**5**	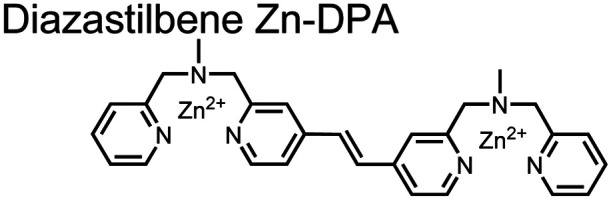	Tau phosphopeptides, proximal pS and/or pT	Fluorescence spectra, ITC, dual emission fluorescence change	50 mM HEPES, pH 7.2		*Chem. Commun.*, 2009, **20**, 2848–2850
**6**	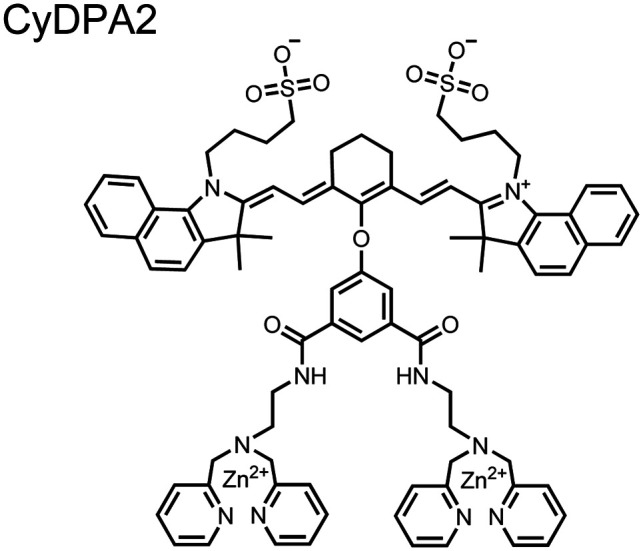	pTau	Ratiometric NIR fluorescence, gel staining, fluorescence microscopy	50 mM HEPES, pH 7.4, 10% DMSO	600 ng mL^−1^ pTau	*Am. J. Nucl. Med. Mol. Imaging*, 2013, **3**(2), 102–117
**7**	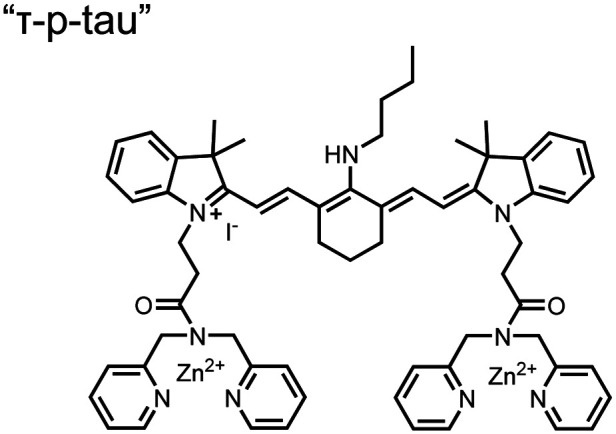	Hyper-phosphorylated Tau	Fluorescence titrations, fluorescence lifetime imaging microscopy (FLIM)	50 mM HEPES, pH 7.5	85 nM	*Anal. Chem.*, 2019, **91**, 3294–3301
**8**	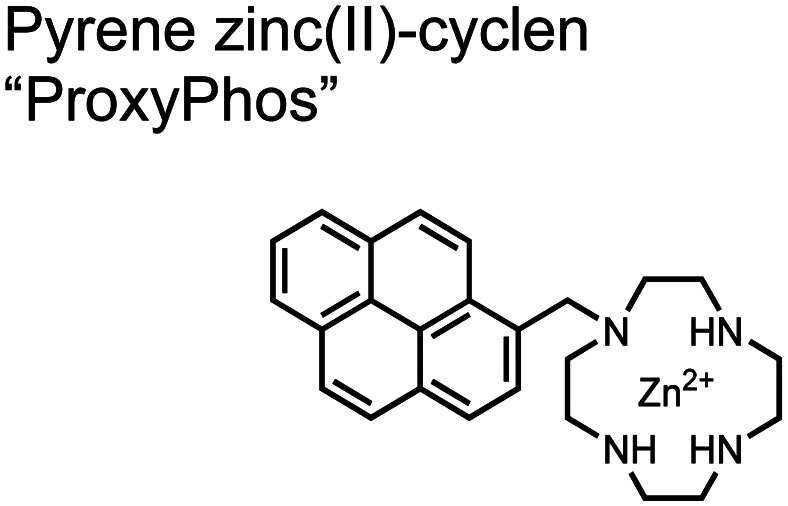	Phosphopeptides containing proximal pS, pY and pT; phosphoproteins including α-casein, β-casein, riboflavin-binding protein and phosvitin	Fluorimeter titrations, stained SDS-PA gels, sequestering experiments, phosphatase enzyme activity titrations	Peptides: 50 mM HEPES, pH 7.5, 10% DMSO; proteins: 50 mM HEPES, pH 7.5, 20% DMSO, 75 mM NaCl	625 nM diphospho-peptides; 80 nM PP-protein (α-casein); 1.2 μg α-casein on PA gel	*J. Am. Chem. Soc.*, 2014, **136**, 1234–1237
						*Analyst*, 2016, **141**, 820–822
						*Analyst*, 2017, **142**, 2451–2459
**9**	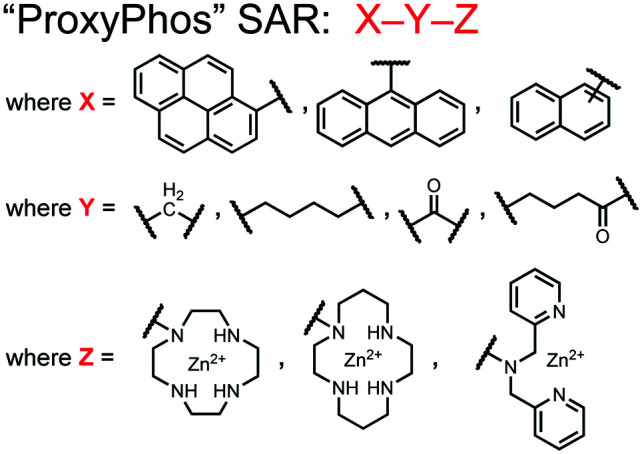	Phosphopeptides containing proximal pS, pY and pT; α-casein, β-casein proteins	Fluorimeter titrations, stained SDS-PA gels, stained mammalian cells	Peptides: 50 mM HEPES, pH 7.5, 10% DMSO; proteins: 50 mM HEPES, pH 7.5, 20% DMSO, 75 mM NaCl	625 nM diphospho-peptides; 80 nM α-casein; 500 ng β-casein on PA gel	*Analyst*, 2017, **142**, 3922–3933

Luminescent chemosensors						
**10**	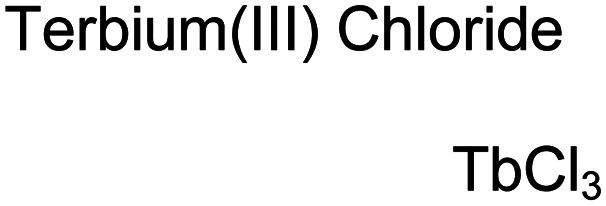	Proximal phosphopeptides where at least one is pY (*i.e.* pTApY, pYpY)	Luminescence spectra/images	50 mM HEPES, 50 mM NaCl, pH 7.5	Microplate reader = 4 μM; UV lamp = 30 μM	*Chem. Commun.*, 2015, **51**, 6675–6677
**11**	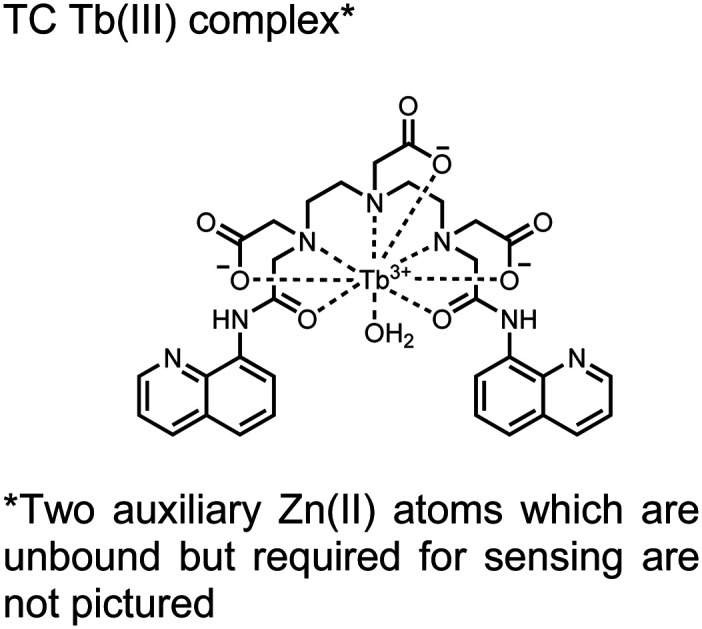	Proximally pSpS phosphorylated tau peptides	Luminescence spectroscopy, time-resolved luminescence spectra	5 mM Tris–HCl, 50 mM NaCl, pH 7.4	62 nM	*Chem. Commun.*, 2015, **51**, 8185–8188
**12**	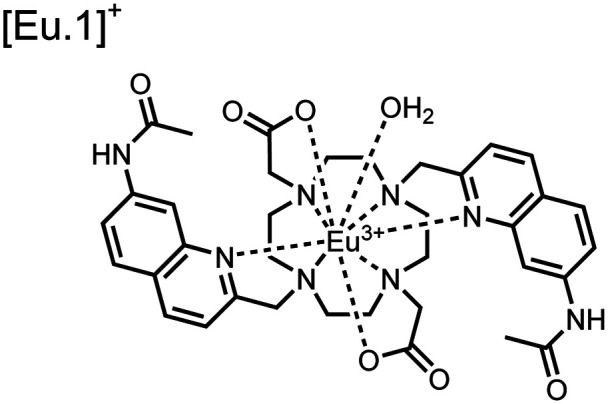	Proximal pYpY phosphopeptides (selective over mono pY, non-phosphorylated Y and proximal pS/pT)	Luminescence titrations, phosphatase catalysed dephosphorylation of pYpY peptides	10 mM HEPES buffer, pH 7.0	1 mM phosphopeptide	*Supramol. Chem.*, 2018, **30**, 765–771

Hybrid biosensors						
**13**	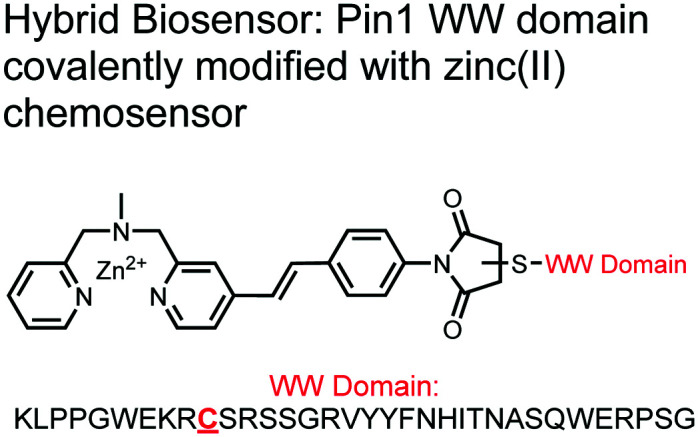	Proximal pS peptides derived from RNA polymerase II CTD	Fluorescence titrations, fluorescence polarization, monitoring of kinase-catalyzed phosphorylation	50 mM HEPES, pH 7.2	1 μM phosphopeptide	*J. Am. Chem. Soc.*, 2007, **129**, 6232–6239
**14**	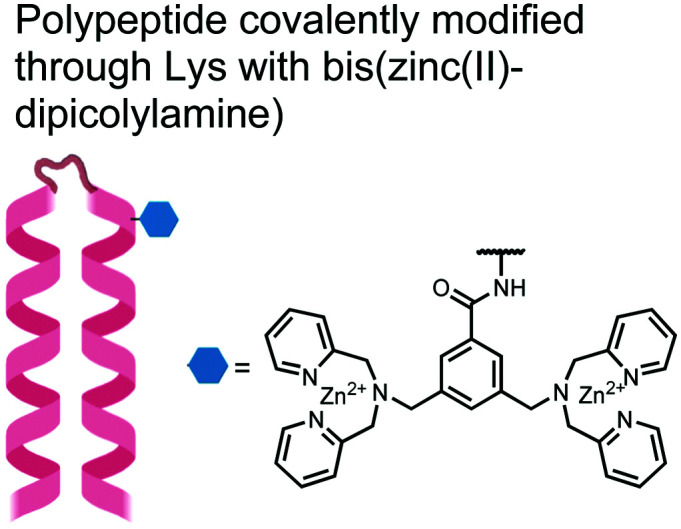	Phosphoproteins including α-casein, β-casein	Fluorescence spectroscopy, pull-down experiments	10 mM HEPES, pH 7.2, 150 mM NaCl	500 nM α-casein by fluorescence, 10 nM α-casein enrichment by pull-down	*Org. Biomol. Chem.*, 2011, **9**, 7697–7704

Inorganic nanoparticles						
**15**	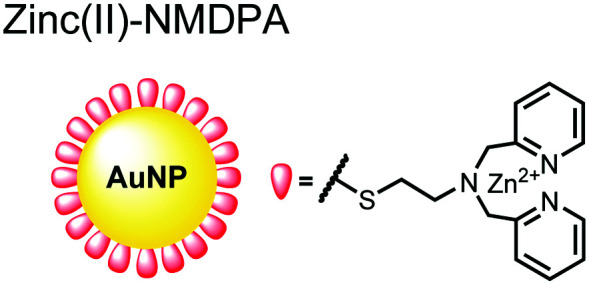	Various pSpS phosphopeptides	Colorimetric detection UV-vis spectroscopy and DC photos	50 mM HEPES, pH 7.2	Naked eye detection at 121 μM	*Sens. Actuators, B*, 2010, **147**, 687–690.
**16**	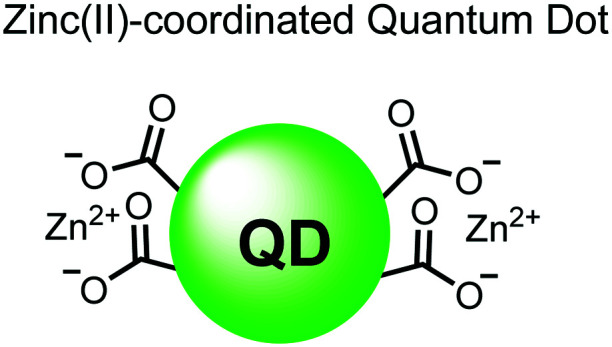	TAMRA-labeled pSpS phosphopeptides	Ratiometric FRET	20 mM Tris–HCl, pH 7.4	80 nM	*Sensors*, 2015, **15**, 17977–17989

## 6. Summary and perspective

**Fig. 7:**
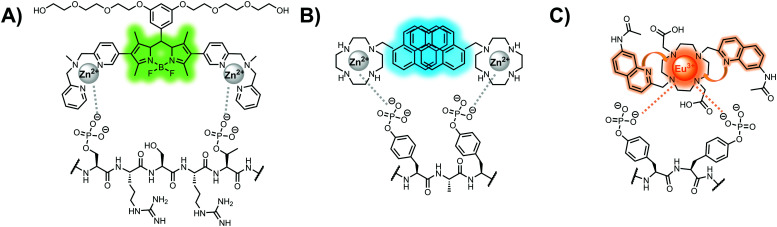
Main binding mechanisms of sensors for proximal phosphorylation. (A) Two or more Zn(ii)-chelate receptors within one sensing scaffold binding to a proximal phosphorylation motif; (B) two individual Zn(ii)-chelate receptor molecules detecting proximal phosphosites with excimer reporting; (C) lanthanide binding to phosphosites with antenna sensitization.

Optical sensing of proximal phosphorylation motifs is a challenging but biologically important field in molecular recognition. To date, the most investigated class of sensors for proximal phosphopeptides and proteins in physiological aqueous conditions is small-molecule fluorescent chemosensors. Initial reports by the Hamachi group focused on using cationic metal ions for complementary binding to anionic phosphate residues. Among metal centers, Zn(ii) has demonstrated the most success, where it has been used to coordinate phosphate residues inspired by natural enzyme receptors. Various metal chelates have been reported, but DPA is by far the most prevalent. In contrast to mono-phosphorylation sensors, detecting proximal phosphosites typically required two or more Zn(ii)-chelate receptors for simultaneous binding to multiple phosphates (Fig. 7A). The positioning of rigid receptors within sensor scaffolds has selectively targeted certain *i*,*i + n* spacings of proximal phosphorylated residues. Although most sensors detect targets in this 1 : 1 fashion, an excimer-based approach that operates *via* a 2 : 1 sensor : analyte mechanism was also investigated (Fig. 7B). Therefore, multiple elegant solutions for binding proximal phosphosites have been designed and reported in the literature, establishing a toolbox of chemosensors applicable to various phosphosite motifs. Advancements in fluorescent reporters have also improved the optical readout of sensors (*i.e.* quantum yield, signal-to-noise). A trend towards increasingly red-shifted fluorophores has allowed for applications beyond fluorimetry including microscopy, SDS-PAGE and histological staining.

Luminescent chemosensors have also been an important modality for detecting proximal phosphosites. Lanthanide ions within metal- or ligand-free complexes have been used as both phosphate receptor and luminescent reporter in optical sensing designs. The requirement for an antenna sensitizer has spatially limited target analytes to proximal motifs that include at least one phosphotyrosine (Fig. 7C). Additionally, spatial limitations of chelated lanthanide ions require proximal motifs with adjacent or near adjacent phosphosites. Time-resolved luminescence and large Stokes shifts were ideal for measuring phosphorylation status in real-time and within complex physiological conditions.

Hybrid biosensors have shown the applicability of combining a biological recognition element with a chemosensory component for proximal phosphosite detection. These conjugate sensors utilize cooperative interactions to improve sequence selectivity and binding potency. However, the desired biological component must be thoroughly investigated for its sequence selectivity, mechanism of binding and appropriate chemical functionalization site. Critically, future designs should also ensure the fixture of a chemical receptor or reporter is robust and does not alter the biological component function. As of now, only short amino acid sequences or small protein domains have been conjugated for hybrid biosensors in the context of proximal phosphorylation. The use of larger, more complex biological components such as whole proteins, antibodies or aptamers might allow for even greater binding affinity and sequence specificity.

The optical properties of inorganic nanoparticles make them great candidates for optical sensors for proximal phosphorylation. The ability to functionalize the nanoparticle surface with a wide assortment of receptors is desirable. However, reports of this class for proximal phosphorylation are lacking. One foreseen challenge is the difficulty of employing selective receptors for proximal phosphates *via* surface chemistry. Unlike small-molecule chemosensors, nanoparticle functionalization has less control over the spacing and rigidity of receptors, which had been shown to be critical in detecting certain *i*,*i + n* phosphate motifs. The current reports have outfitted nanoparticles with metal chelates but suffer from off-target binding. The use of inorganic nanoparticles for proximal phosphorylation is challenging, but superior optical properties could pivot towards the solution for sensing in complex biological applications.

Among these reports, model proximally phosphorylated peptides were the prime analytes of interest, with some being derived from biologically relevant proteins such as RNA polymerase II and heat shock factor 1. Although useful study tools, a model proximally phosphorylated peptide of *i*,*i + n* spacing may not be entirely representative of the same motif in the parent protein due to the presence or absence of secondary structures. The structures of model peptides should be validated by other analytical methods such as CD spectroscopy prior to study if selectivity for a certain *i*,*i* + *n* spacing is desired, although this is likely less critical with IDPs and hyperphosphorylated aggregate proteins. When applicable, direct detection of the proximally phosphorylated protein of interest is preferred to avoid these differences. Detection of proximally hyper-phosphorylated Tau protein and its NFTs has been a major research interest due to the broad scope of clinical implications. Optical chemosensors have been able to preferentially detect pTau down to double-digit nanomolar concentrations in a variety of assay types. Enzymatic reactions have also been assayed *in vitro*, including kinases (*i.e.* GSK3β) and phosphatases. The ability to tailor optical sensors with sequence selectivity towards proximal phosphorylation motifs over mono- and non-phosphorylated analogues will allow for the design of facile and robust screening assays for enzyme activity, drug inhibition or activation and probing downstream physiological effects.

The identification of proximal multisite phosphorylation is growing increasingly important in protein biology with an increasing emphasis on translational applications. Although there has been success with a variety of chemotypes, novel receptors beyond metal-chelates that are tailored to specific applications are crucial to expand the list of tractable targets. The field is looking forward to innovative sensor designs and applications to thoroughly investigate this important protein modification.

## Conflicts of interest

There are no conflicts to declare.

## Supplementary Material
